# Validation of semi-automated flow-mediated dilation measurement in healthy volunteers

**DOI:** 10.1097/MBP.0000000000000448

**Published:** 2020-05-11

**Authors:** Laurence J. Dobbie, Sharon T. Mackin, Katrina Hogarth, Frances Lonergan, Dennis Kannenkeril, Katriona Brooksbank, Christian Delles

**Affiliations:** a Institute of Cardiovascular and Medical Sciences, University of Glasgow, Glasgow, UK; b Department of Nephrology and Hypertension, University of Erlangen-Nürnberg, Germany

**Keywords:** cardiovascular risk, clinical research, endothelial function, flow-mediated dilation

## Abstract

Flow-mediated dilation (FMD) is a non-invasive imaging modality used to measure endothelial function but has significant intra- and inter-observer variability. The use of semi-automated FMD devices could overcome this limitation. We assessed the reproducibility of same-day semi-automated FMD measurements by investigators who received basic training on the correct use of the device.

**Methods:**

Forty-three healthy volunteers had two brachial artery FMD measurements performed 20 minutes apart using the UNEX EF 38G device, and automated outputs were produced. Images were also manually analysed using edge-detection software. The reproducibility of repeat FMD measurements within individuals was compared for automated and manual readings, and the correlation between analytical techniques was calculated.

**Results:**

Twenty-five percent of scans were of non-diagnostic quality (n = 32). Automated analyses demonstrated sub-optimal reproducibility and measurement variability [intraclass correlation coefficient (ICCC) = 0.334, coefficient of variation (CV) = 45.87%]. In contrast, manually analysed scans had excellent reproducibility and low measurement variance (ICCC = 0.815, CV = 11.40%). FMD values obtained from automated and manual analysis correlated poorly (r = 0.164), whereas resting (r = 0.955) and maximal brachial artery diameters demonstrated excellent correlation (r = 0.867).

**Conclusion:**

Manually evaluated serial UNEX EF readings have good reproducibility and therefore, the optimal FMD workflow involves manual analyses prior to independent automated interrogation. The high non-diagnostic scan rate is most likely the result of insufficient training and indicates that semi-automatic devices such as UNEX EF should be used by experienced investigators to achieve optimal results.

## Abstract 2

Supplemental Digital Content is available in the text.

## Introduction

In the healthy vasculature, vascular tone is modulated by endothelium-derived vasoactive molecules which either cause relaxation or constriction of vessels [1]. The critical vasodilator nitric oxide (NO) is released by endothelial cells in response to various stimuli including shear stress due to circulating blood. Reduced NO bioavailability, for example, as a result of oxidative stress, leads to impaired vasodilation or inappropriate vasoconstriction which indicate endothelial dysfunction [2,3]. Endothelial dysfunction is a key early event in atherosclerosis which can eventually lead to cardiovascular disease (CVD). Due to the endothelium’s pathological significance, techniques have been developed to measure its function *in vivo*. However, these methods must be proven to be both robust and tolerated by patients [4].

Endothelial function can be measured by several non-invasive methods. The current gold standard is brachial artery flow-mediated dilation (FMD) which quantifies NO-dependent arterial vasodilation [5,6]. FMD is a technique that has been used for over three decades in clinical research to measure arterial function *in vivo* as percentage dilation of the brachial artery after a period of forearm occlusion [7]. The intraluminal artery diameter is measured via an ultrasound probe and is used to calculate a percentage FMD (% FMD). FMD has been shown to predict cardiovascular events and hence has potential utility as an index of CVD risk and progression [8,9]. Yet, this technique is limited by requiring extensive operator training and having significant inter and intra-user variability. This is due to the technique’s sensitivity to differences in ultrasound probe position, participant preparation, cuff occlusion time and image acquisition, meaning extensive standardisation is required [7]. For instance, participants should abstain from caffeine and alcohol for at least 4 hours prior to measurement to minimise the impact of confounding factors. These difficulties are further compounded by the inherent biological variability of endothelial function. Thus, the production of a semi-automated system that uses the same protocol at every measurement could minimise the inter-operator error and hence the measurement variability [10,11].

A recently developed semi-automated solution, the UNEX EF (UNEX Corporation, Nagoya, Japan), is now also available in Europe [12]. The device was validated in a Japanese population by comparing measurement variability over multiple sites. The investigators demonstrated that semi-automated FMD has an acceptable reproducibility in cases of a clear recording [13]. Yet, this study did not interrogate same-day measurement reproducibility, included participants with varying degrees of cardiovascular health and failed to compare machine and operator generated results. Thus, there is currently no published evidence evaluating the reproducibility of same-day UNEX EF measurements. It is also important to validate semi-automated FMD in Caucasians as they have a different body habitus compared to the Japanese which may result in technical challenges for tracking the brachial artery.

We, therefore, aimed to validate the UNEX EF as a method to measure FMD in a healthy Caucasian cohort. We hypothesised that the device provides a reproducible measure of endothelial function in healthy volunteers.

## Methods

### Participants and study design

Healthy volunteers (n = 43) were recruited from the University of Glasgow and provided written informed consent before recruitment. Inclusion criteria were age between 18 and 40 years and absence of overt cardiovascular or other disease such as hypertension, renal disease, previous cardiovascular event, previous cancer, heart failure and hyperlipidaemia. Pregnant women were not included. Demographic and medical history information was collected by use of a structured case report form. Volunteers attended a study visit and had two FMD measurements performed 20 minutes apart. The recruits were asked to abstain from caffeine and nicotine products for at least 4 hours prior to their visit. The participants returned for a second study visit undergoing the same protocol 1–19 days after the first visit.

The study’s primary aim was reproducibility, that is, comparison between two measurements on the same day. The secondary aim was to study biological variability, that is, comparison of the mean of measurements between two study days. Aim 1 was addressed by analysing scans from day 1. For some participants (n = 6), two valid readings could not be obtained on day 1 and therefore two valid readings from day 2 were used to address the primary aim.

The study was approved by the University of Glasgow College of Medical, Veterinary & Life Sciences Ethics Committee for Non-Clinical Research Involving Human Subjects (application no 200170184).

### Flow-mediated dilation protocol and image analysis

The UNEX EF is a novel semi-automated FMD device that utilises B-mode ultrasound by capturing one long-axis and two short-axis images. The long-axis image provides a longitudinal view whereas the two short-axis arrays provide a cross-sectional brachial artery view. The probe is attached to a hybrid arm which automatically tracks the arrays. When the three images are collated this facilitates accurate probe positioning with continuous correction [12].

Participants lay on a bed in a quiet, temperature-controlled room (~23°C) for at least 5 minutes to obtain a resting blood pressure (BP) using a standard sphygmomanometer on their left arm. An occlusion cuff was placed around the right forearm and two ECG leads were attached to the wrists. The ultrasound probe was placed 5-10 cm proximal to the elbow with a probe holder ensuring image consistency. A tracking gate measured the brachial artery’s rest diameter from the computer assessed intima-intima. The occlusion cuff was inflated 50 mmHg above SBP for 5 minutes. Following 5 minutes of forearm ischaemia, the cuff was deflated, and the brachial artery was tracked for 2 minutes. Automated outputs generated by the UNEX EF for the rest diameter, maximum diameter and % FMD were recorded. The UNEX EF calculated % FMD as [(Max Diameter − Rest Diameter)/Rest Diameter] × 100. Supplementary Figure 1, Supplemental digital content 1, http://links.lww.com/BPMJ/A117 outlines the FMD protocol [14].

Images were later manually analysed by a single operator using the UNEX EF PC analysis software (UNEX Corporation). This acted like a quality control whereby scan diameters were optimised and non-diagnostic scans were eliminated. Scans were deemed non-diagnostic if the intima-intima could not be traced throughout the reading or if no blood flow was detected. The brachial artery diameters were fine-tuned as the maximum and/or rest diameters were incorrect in certain instances.

### Statistical analysis

Reproducibility of automated and manual measurements was interrogated via the coefficient of variation (CV) and the intraclass correlation coefficient (ICCC; two-way mixed-effects model based on the absolute agreement of single measures). The agreement between analytical methods was assessed via Pearson correlation and ICCC (two-way mixed-effects model based on the absolute agreement of average measures) [15]. Bland–Altman plots were used to study bias between measurements. Data are provided as mean ± SD unless otherwise stated. Analyses were conducted using SPSS (version 23.0; IBM, New York, New York, USA).

## Results

### Study participants

Forty-three volunteers took part in the study. Of these, only 32 could be analysed due to a 25.3% non-diagnostic scan rate. The clinical characteristics and demographics of this final cohort are reported in Table 1. Information on the whole cohort is reported in Supplementary Table 1, Supplemental digital content 1, http://links.lww.com/BPMJ/A117.

**Table 1:**
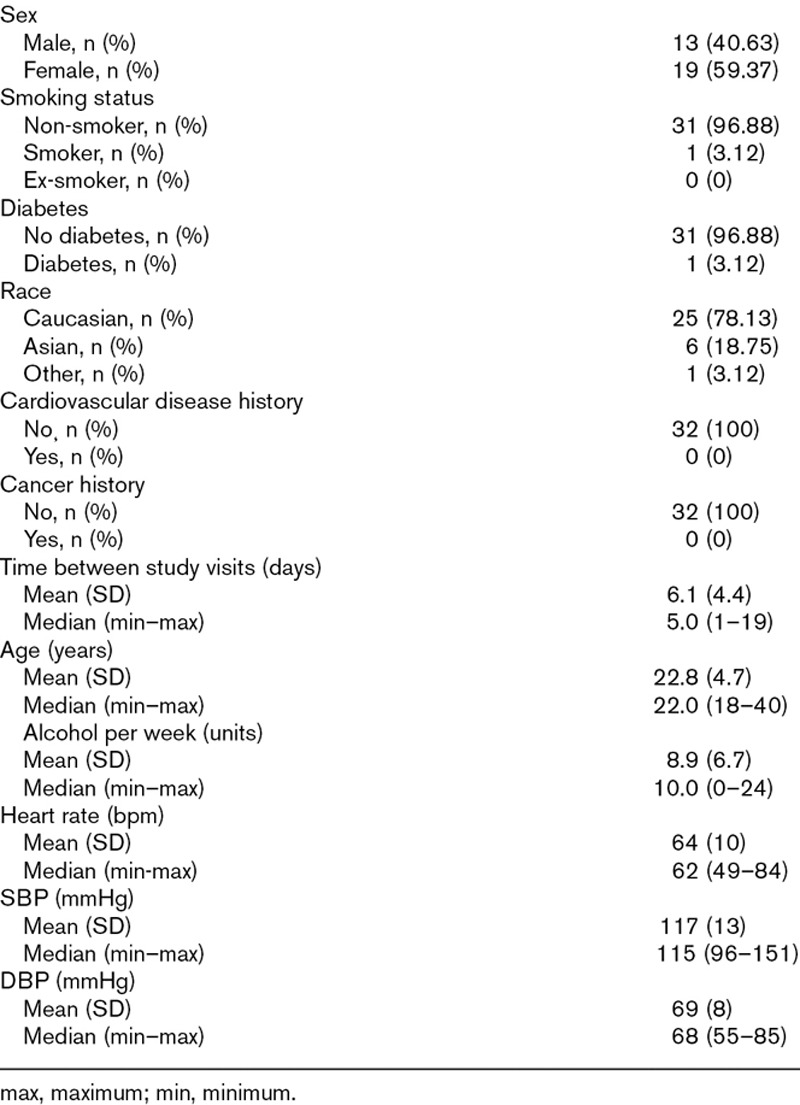
Participant characteristics

### Same day measurements: automated analysis

We first employed the automated data analysis feature of the UNEX-EF device.

On automated analysis, the mean measurement 1 brachial artery diameter was 3.32 ± 0.60 mm at rest and 3.58 ± 0.59 mm at maximal dilation, corresponding to an FMD of 8.57 ± 6.54% (Table 2). The mean measurement 2 brachial artery diameter was 3.28 ± 0.59 mm at baseline and 3.55 ± 0.65 mm at maximal dilation, corresponding to an FMD of 7.99 ± 4.90%.

**Table 2:**
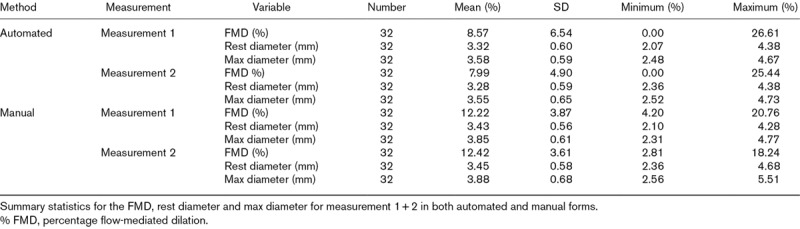
Summary statistics of flow-mediated dilation measurements

**Fig. 1:**
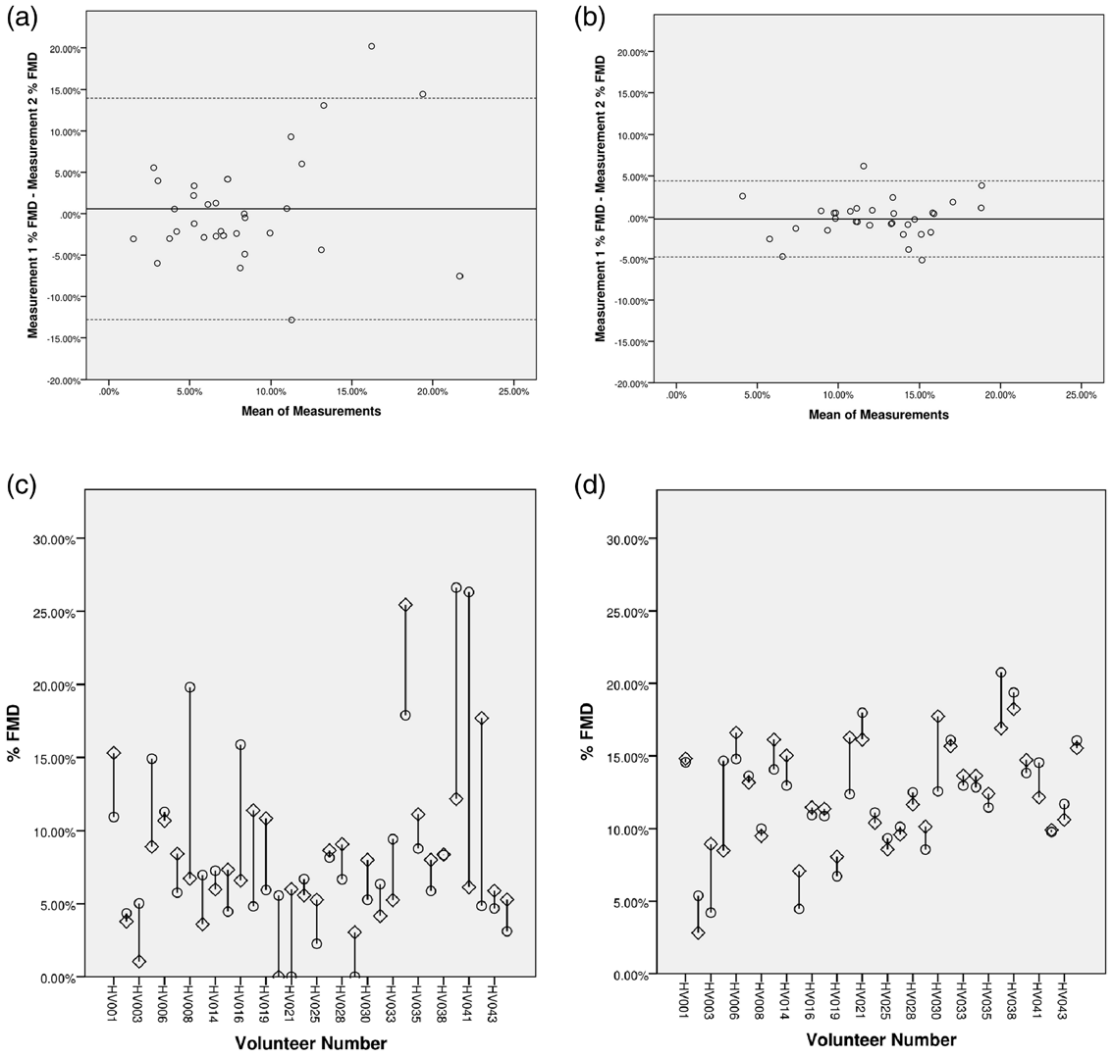
Bland–Altman plots and scatterplots of % FMD for automated and manual Results. (a and B) Reproducibility of FMD for automated and manual analyses, illustrated by means of Bland–Altman analysis. (c and d) Scatterplots showing the % FMD for measurement 1 and 2. (a) Automated results Bland–Altman plot; (b) manual results Bland–Altman plot; (c) automated results scatterplot; (d) manual results scatterplot; open circles = measurement 1; open diamonds = measurement 2; broken lines = 95% limits of agreement. % FMD = percentage flow-mediated dilation; HV, healthy volunteer.

Table 3 details the reproducibility results. Repeat measurements had poor reproducibility [ICCC: 0.334 (−0.016 to 0.610)] and high measurement variability (CV: 45.87%). A Bland–Altman plot examined the difference between measurements 1 and 2 (Fig. 1a). This demonstrated poor reproducibility as shown by the wide 95% limits of agreement (Table 3: −12.8 to 13.96%). Two subjects did not fall within the 95% limits of agreement. There was no systemic bias between measurements.

**Table 3:**
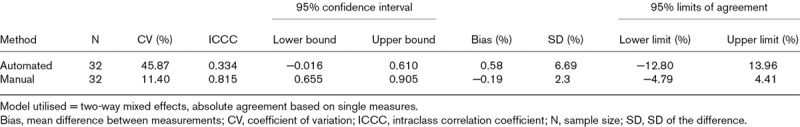
Reproducibility statistics

### Same day measurements: manual analysis

We then analysed the data manually with the UNEX-EF software.

On manual analysis, the mean measurement 1 diameter was 3.43 ± 0.56 mm at baseline and 3.85 ± 0.61 mm at maximal dilation, corresponding to an FMD of 12.22 ± 3.87% (Table 2). The mean measurement 2 diameter was 3.45 ± 0.58 mm at rest and 3.88 ± 0.68 mm at maximal dilatation, corresponding to an FMD of 12.42 ± 3.61%.

Manual analyses demonstrated good reproducibility for repeat measurements [ICCC: 0.815 (0.655–0.905)] with low measurement variability (Table 3, CV: 11.40%). A Bland–Altman plot showed acceptable reproducibility as highlighted by the relatively tight 95% limits of agreement (Fig. 1b and Table 3: −4.79 to 4.41%). Two subjects did not fall within the 95% limits of agreement. There was no systemic bias between measurements.

### Correlation between automated and manual findings

**Fig. 2:**
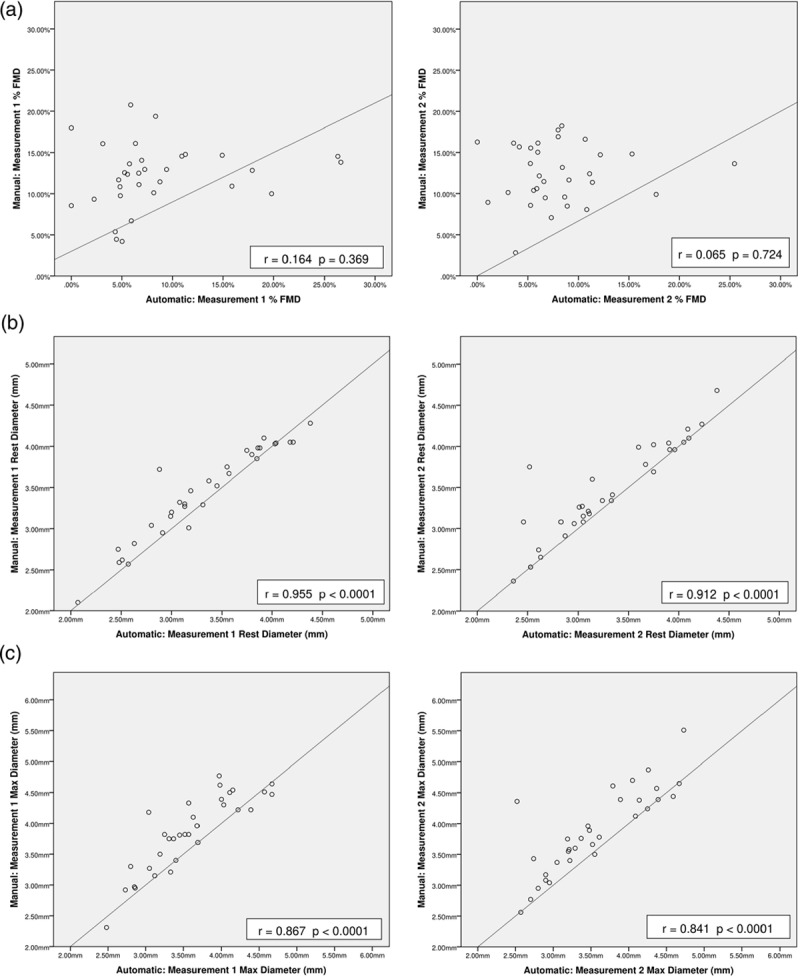
Automated versus manual analyses scatterplots. Scatterplots of manual versus automated results. (a) Measurement 1 + 2 % FMD Scatterplots; (b) measurement 1 + 2 rest diameter scatterplots; (c) measurement 1 + 2 max diameter scatterplots. The line of best fit is displayed in each graph; r = Pearson correlation coefficient; % FMD = percentage flow-mediated dilation.

Subjectively, as demonstrated by the scatterplots, data variability was lower in manual compared to automated analyses (Fig. 1c and d). The closeness of manual and automated measurements was assessed by correlation and further scatterplots (Table 4 and Fig. 2). Pearson correlation coefficients for % FMD were 0.164 (*P* = 0.369) for measurement 1 and 0.065 (*P* = 0.724) for measurement 2 (Table 4). The rest diameter’s correlation coefficients were 0.955 (*P* < 0.0001) for measurement 1 and 0.912 (*P* < 0.0001) for measurement 2. The maximum diameter’s correlation coefficients were 0.867 (*P* < 0.0001) and 0.841 (*P* < 0.0001) for measurements 1 and 2, respectively.

**Table 4:**
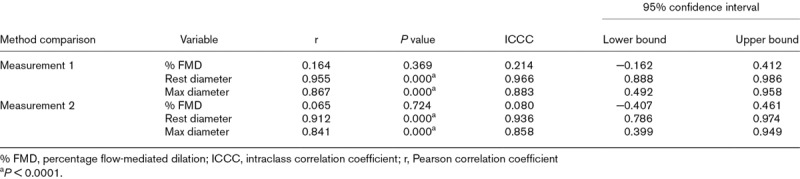
Correlation statistics

Formally, reproducibility of automated and manual analyses was assessed by ICCC (Table 4). The reproducibility of % FMD was poor for measurement 1 [ICCC: 0.214 (−0.162 to 0.412)] and measurement 2 [ICCC: 0.080 (−0.407 to 0.461)]. The rest diameter’s measurement reproducibility was excellent for measurement 1 [ICCC: 0.966 (0.888–0.986)] and measurement 2 [ICCC: 0.936 (0.786–0.974)]. Similarly, the maximum diameter’s reproducibility was good for measurement 1 [ICCC: 0.883 (0.492–0.958)] and measurement 2 [ICCC: 0.858 (0.399–0.949)].

### Additional data

Between days there was acceptable reproducibility and a low measurement variance for manual analyses [Supplementary Table 2, Supplemental digital content 1, http://links.lww.com/BPMJ/A117: ICCC: 0.679 (0.431–0.833); CV: 12.43%]. A sensitivity analysis involving exclusion of smokers and individuals with diabetes did not affect the reproducibility of readings (Supplementary Table 3, Supplemental digital content 1, http://links.lww.com/BPMJ/A117). Subgroup analyses of sex, SBP and DBP did not affect the reproducibility of measurements. (Supplementary Table 4, Supplemental digital content 1, http://links.lww.com/BPMJ/A117). Supplementary Bland–Altman plots examine the reproducibility of automatic and manual results for % FMD, rest diameter and maximum diameter (Supplementary Figures 2–4, Supplemental digital content 1, http://links.lww.com/BPMJ/A117). The reproducibility statistics which were used to produce the Bland–Altman plots are presented in supplementary Table 5, Supplemental digital content 1, http://links.lww.com/BPMJ/A117.

## Discussion

This study determined the within-day variability of FMD assessed by the UNEX EF device in healthy volunteers. We found manually derived measurements to have good reproducibility and low variability. To the best of our knowledge, this study is unique as it examines the reproducibility of the UNEX EF’s readings in a young healthy Caucasian cohort. The only other UNEX EF validation study reported an acceptable inter-user reproducibility in Japanese individuals (ICCC: 0.862) [13]. This coefficient was based on the findings of multiple centres – with some centres reporting sub-optimal reproducibility. Additionally, this previous study was not based on same-day repeat measurements. Thus, this data cannot be directly compared with data obtained from our Caucasian cohort.

Our results demonstrate poor correlation between data obtained from automated and manual analyses. We found that the device intermittently tracks a false tunica intima, which translated into incorrect data, poor reproducibility and consequently sub-optimal concordance with manually analysed data. Yet, the excellent correlation between the rest and maximum diameters for separate automated measurements demonstrates that the same blood vessel is being interrogated at each measurement. Thus, the variation is due to measurement error whereby very small differences in vessel diameter measurements translate into larger % FMD changes.

A Bland–Altman plot for automated measurements showed poor reproducibility with the 95% limits of agreement ranging from −12.8 to 13.96%. Yet, most of the repeat readings fell within 5% of each other. The wide interval is partly due to significant differences in repeat measurements which tended to occur at higher FMD values. This could indicate that the device’s reproducibility decreases as the % FMD increases. For manual results whilst the 95% limits of agreement ranged from −4.79 to 4.41%, most measurements were within 2% of each other. This indicates good reproducibility, which is in keeping with our other findings. Additionally, unlike automated measurements, the difference between readings was not affected by the % FMD.

The data reported echoes the findings of other FMD validation studies. A moderately sized trial (n = 38, of which 18 participants had established CVD) by Onkelinx *et al.* [11] demonstrated that same day FMD measurements had excellent reproducibility (ICCC: 0.94). Another larger study (n = 109) by van Mil *et al.* [16] reported a 9.3% CV in a healthy cohort. The superior reproducibility reported by these studies could be explained by operators having undergone extensive training. Additionally, the study by van Mil *et al.* [16] recruited older individuals (mean age = 46 years), which may represent a group with a different haemodynamic response to our younger cohort [17].

This study informs the future use of the UNEX EF device. Manual analyses were shown to be superior to automated results, and there was a non-diagnostic scan rate of 25% picked up on manual review of images. These findings have two short-term implications for the UNEX EF. First, following measurements, recorded scans should be reviewed as part of a quality control protocol. If deemed inadequate the scan should be repeated. Second, all readings require manual evaluation to ensure an optimal measure of brachial artery diameter. Together, these methods will ensure scan reproducibility and hence minimise measurement error.

Our investigators only underwent limited and basic training in the correct use of the UNEX EF device. This approach was deliberately taken in order to test whether automated artery tracking and FMD analysis produces correct data if used by inexperienced investigators. We strongly advocate for more extensive training, familiarisation with all of the device’s features and manual cross-checking of data before performing assessments in a research context. More experienced users may find less need for confirmatory manual analysis.

Importantly, this study does not imply that automated analyses cannot be used in research projects and potentially in future clinical applications. There is currently no data to inform power calculations for clinical research utilising the UNEX EF. As previously discussed, the only other validation study had limited external validity when comparing it to a Caucasian population. Thus, our automated and manual results will inform power calculations to determine the sample size for future studies.

Manually evaluated same-day measurements had good reproducibility whereas readings on different days had moderate reproducibility. This difference is most likely attributed to the biological variability in endothelial function occurring between days. Thus, the disparity likely reflects FMD’s dynamic nature rather than machine error.

The study population was healthy apart from one smoker and one diabetic individual and none of the participants had any overt CVD. These covariates are thought to increase FMD variability due to their importance in the pathogenesis of atherosclerosis, although Ibrahimi *et al.* [6] found that the ICCC for smokers and non-smokers was equivalent. Whilst our study was not designed to formally test the effect of these CVD risk factors it is reassuring that exclusion of the two participants who smoked or had diabetes did not change the results.

### Strengths and limitations

The study itself had several strengths. First, the population interrogated was largely homogenous. Therefore, the observed variability is unlikely to be due to differences in clinical parameters. Second, measurements were taken in quick succession on the same day. Thus, it is less probable that physiological variability impacted the documented reproducibility. The study’s main weakness is the small sample size. This impacted the certainty in the reproducibility assessment as reflected by the relatively wide ICCC confidence intervals. The study size also limited the subgroup analyses of smoking and diabetes. However, the sample size is comparable to that of other FMD validation studies. For instance, the FMD validation study by Ibrahimi *et al.* [6] recruited fewer participants (n = 27). Furthermore, much of the literature includes participants with overt CVD, whereas our study utilised a young healthy cohort.

FMD is hampered by its sensitivity to methodology variation. The UNEX EF’s semi-automated process overcomes many factors known to reduce the technique’s reproducibility. Primitive FMD used an arbitrary peak diameter (i.e. at 60 seconds) which regularly produced misleading conclusions as the time to maximum diameter varies considerably. The UNEX EF continuously measures the brachial artery allowing calculation of the true peak diameter [18]. However, when the scan quality is deemed inadequate by the UNEX EF device, it reverts to automated FMD calculations from short-axis views rather than longitudinal. If there was a skew on the short axis, this may have not been comparable to the longitudinal FMD. The device was further limited by the software intermittently tracking other structures like tendons or veins which reduced repeatability of data obtained from automated analysis. Yet, these problems are easily corrected with manual analysis.

Traditional FMD requires 3–6 months of training to ensure measurement consistency. In comparison, the UNEX EF requires less intensive training to obtain adequate readings. Additionally, compared to traditional FMD, less work is required to check inter-user variability due to the technique’s semi-automated nature. We appreciate, however, that the minimal training that investigators in the present study received was not sufficient to generate optimal results. This led to a relatively large number of non-diagnostic scans and a suboptimal image quality that made automated analysis more difficult and unreliable.

The operator conducting the manual analysis was not blinded to the previous results. This could have biased the findings. Yet, the improved reproducibility is likely due to error correction rather than analysis bias. This is because the software tracked a false tunica-intima in certain instances, with the operator applying a post-hoc adjustment to facilitate accurate measurement. These adjustments were minor as shown by the excellent correlation between the rest and maximum diameters. The poor correlation between percentage FMD is due to very small differences in diameter, as little as 0.1 mm, having a significant effect on the output. Hence, the improved manual reproducibility is likely to highlight the optimal FMD workflow rather than operator bias.

A general limitation of FMD is that it fails to measure the pathologically significant coronary arteries. Directly measuring these arteries – via quantitative coronary angiography – gives a clear interpretation of atherosclerosis progression. Yet, % FMD does correlate with coronary artery endothelial function and is non-invasive making it the advantageous technique in most instances [5].

### Conclusion

We evaluated a novel semi-automatic device for the measurement of FMD. In our study, data obtained from manual image analysis had good reproducibility whereas data obtained from automated analysis had poor reproducibility. We also found a significant rate of non-diagnostic scans. Taken together the optimal workflow of studies using the UNEX EF device should include manual quality assessment and analysis. We also strongly recommend intensive training of operators. This is because the automated edge detection and the ultrasound probe positioning only work reliably when study participants are optimally prepared and the probe is correctly placed. The study also highlights that further progress is required before FMD can be used clinically although semi-automated devices such as UNEX EF are clearly a step in the right direction.

## Acknowledgements

We thank the participants for kindly volunteering to help with this study. We would also like to thank Dr. John Harris who kindly provided us with space to work within the Teaching and Learning Centre, Queen Elizabeth University Hospital.

C.D. and K.B. are supported by a British Heart Foundation Centre of Research Excellence Award (RE/13/5/30177 and RE/18/6/34217). S.T.M. is funded by the Glasgow Children’s Hospital Charity.

## Conflicts of interest

There are no conflicts of interest.

## Supplementary Material


