# Supplementary material for: Validation of semi-automated flow-mediated dilation measurement in healthy volunteers
Source: Blood Press Monit. 2020 May 11;25(4):216–23. doi: 10.1097/MBP.0000000000000448 (PMC7331823; doi:10.1097/MBP.0000000000000448)
Supplement: Supplementary file 1 [file bpmj-25-216-s001.pdf]

Validation of Semi-automated Flow Mediated Dilation Measurement In Healthy Volunteers

Short title: Semi-automated flow mediated dilation measurement

Laurence J Dobbie[1], Sharon T Mackin[1], Katrina Hogarth[1], Frances Lonergan[1], Dennis Kannenkeril[2], Katriona Brooksbank[1], Christian Delles[1]

[1] Institute of Cardiovascular and Medical Sciences, University of Glasgow, Glasgow, UK

[2] Department of Nephrology and Hypertension, University of Erlangen-Nürnberg, Germany

Supplementary Material

Supplementary Table 1: Whole Cohort Characteristics

**Sex**	
Male, n (%)	18 (41.9)
Female, n (%)	25 (58.1)
**Smoking Status**	
Non-smoker, n (%)	39 (90.7)
Smoker, n (%)	1 (2.3)
Ex-smoker, n (%)	3 (7.0)
**Diabetes**	
No diabetes, n (%)	41 (95.4)
Diabetes, n (%)	2 (4.7)
**Race**	
Caucasian, n (%)	35 (81.4)
Asian, n (%)	6 (14.0)
Other, n (%)	2 (4.7)
**Cardiovascular Disease History**	
No, n (%)	43 (100)
Yes, n (%)	0 (0)
**Cancer History**	
No, n (%)	43 (100)
Yes, n (%)	0 (0)
**Time Between Study Visits (Days)**	
Mean (SD)	6.2 (4.2)
Median (min – max)	6.0 (1 – 19)
**Age (Years)**	
Mean (SD)	22.3 (4.2)
Median (min – max)	22.0 (18 – 40)
**Alcohol per week (units)**	
Mean (SD)	9.1 (7.0)
Median (min – max)	7.0 (0.0 – 30.0)
**Heart Rate (bpm)**	
Mean (SD)	66 (10)
Median (min-max)	67 (49 – 84)
**Systolic Blood Pressure (mmHg)**	
Mean (SD)	116 (13)
Median (min – max)	114 (93 – 151)
**Diastolic Blood Pressure (mmHg)**	
Mean (SD)	69 (8)
Median (min – max)	68 (54 – 86)

SD = standard deviation, min = minimum, max = maximum. A two-way t-test demonstrated there was no significant difference in characteristics between the supplement and main study cohort.

Supplementary Table 2: Reliability Statistics between different days

Measurement	N	CV	ICCC	95 % Confidence Interval	
				Lower Bound	Upper Bound
**Day 1 mean FMD + Day 2 measurement 1 FMD**	30	12.43%	0.679	0.431	0.833

N = sample size, CV = Coefficient of Variation, ICCC = intraclass correlation coefficient. Model utilised = two-way mixed effects, absolute agreement based on single measures.

Supplementary Table 3: Sensitivity Analyses on elimination of diabetics and smokers

	N	ICCC	95% Confidence Interval	
			Lower Bound	Upper Bound
**Day 1 Measurements**	30	0.810	0.638	0.905

ICCC = intraclass correlation coefficient, N = sample size. Sensitivity analyses on elimination of one diabetic and one smoker

Supplementary Table 4: Subgroup Analyses of Sex, systolic blood pressure and diastolic blood pressure

Variable		N	Measurement 1: Mean FMD % ± SD	Measurement 2: Mean FMD % ± SD	ICCC	95% Confidence Interval	
						Lower Bound	Upper Bound
**Sex**	**Male**	13	12.06 ± 5.17	12.61 ± 4.53	0.839	0.562	0.948
	**Female**	19	12.33 ± 2.83	12.29 ± 2.96	0.786	0.522	0.912
**Systolic Blood Pressure Median = 115**	**<Median**	16	12.08 ± 2.69	12.41 ± 3.14	0.744	0.425	0.899
	**>Median**	16	12.35 ± 4.85	12.42 ± 4.10	0.856	0.634	0.947
**Diastolic Blood Pressure Median = 68**	**<Median**	16	12.12 ± 3.02	12.41 ± 3.24	0.737	0.376	0.903
	**>Median**	16	12.27 ± 4.74	12.34 ± 4.14	0.859	0.641	0.949

N = sample size, FMD = Flow Mediated Dilatation, ICCC = intraclass correlation coefficient. Subgroup analyses of sex, systolic blood pressure and diastolic blood pressure. Model utilised = two-way mixed effects, absolute agreement based on single measures.

Supplementary Table 5: Supplementary Reproducibility Statistics

Measurement Method	Variable	Within or Between Day Reproducibility	Bias	SD	95% Limits of Agreement	
					Lower Limit (%)	Upper Limit (%)
**Automatic**	**FMD**	**Between**	-1.151%	10.204%	-21.14	18.84
	**Rest Diameter**	**Within**	0.033 mm	0.386 mm	-0.727	0.793
		**Between**	-.0537 mm	0.40 mm	-0.838	0.730
	**Max Diameter**	**Within**	0.0375 mm	0.423 mm	-0.786	0.861
		**Between**	-0.1137 mm	0.493 mm	-1.05	0.823
**Manual**	**FMD**	**Between**	-0.597%	3.241%	-6.947	5.753
	**Rest Diameter**	**Within**	-0.012 mm	0.361 mm	-0.720	0.696
		**Between**	-0.034 mm	0.389 mm	-0.796	0.728
	**Max Diameter**	**Within**	-0.0263 mm	0.405 mm	-0.820	0.768
		**Between**	-0.0627 mm	0.408 mm	-0.879	0.753

FMD = flow mediated dilation, SD = standard deviation, max = maximum

Supplementary Figure 1: FMD Protocol

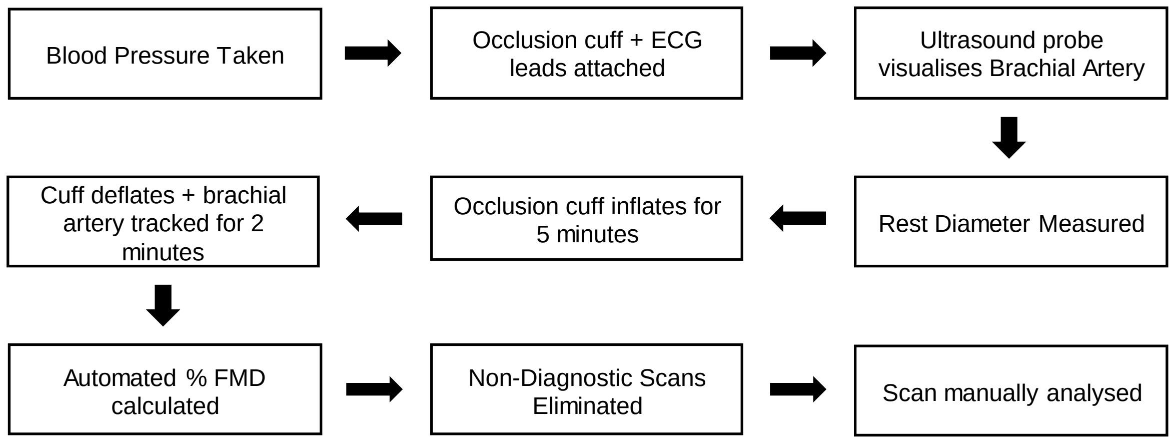

% FMD = percentage flow mediated dilation.

Supplementary Figure 2: Bland-Altman Plots of % FMD

A

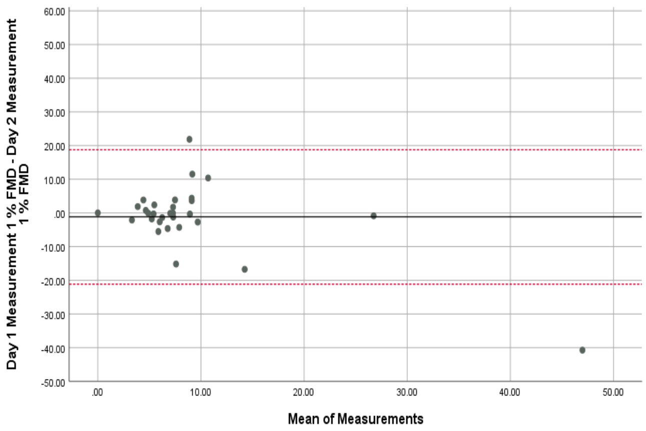

B

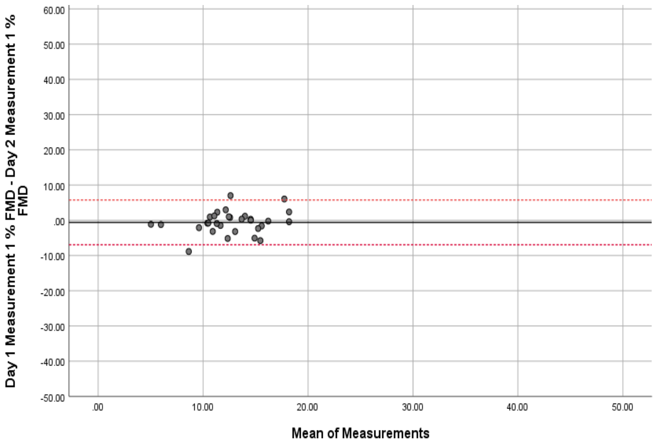

Bland-Altman plots of % FMD readings conducted on separate days for manual and automated analysis. A: Automated results FMD on separate days, B: Manual results FMD on separate days, Broken red lines = 95% limits of agreement, % FMD = percentage flow mediated dilation

Supplementary Figure 3: Bland-Altman Plots of Rest Diameters

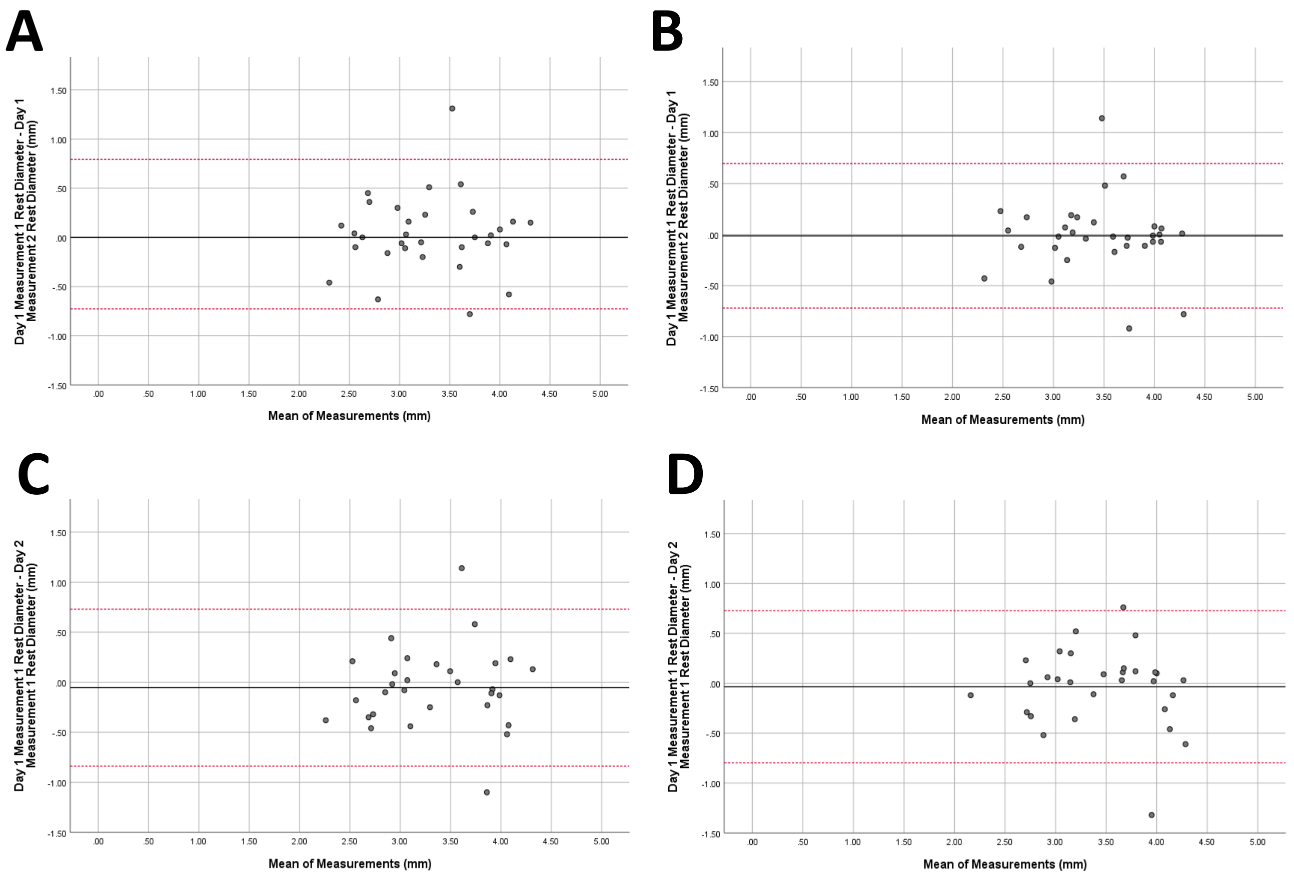

Bland-Altman plots of rest diameters for manual and automated analysis. A: Automated results rest diameter on the same day, B: Manual Results rest diameter on the same day, C: Automated results rest diameter on separate days, D: Manual results rest diameter on separate days, Broken red lines = 95% limits of agreement, % FMD = percentage flow mediated dilation

Supplementary Figure 4: Bland-Altman Plots of Maximum Diameters

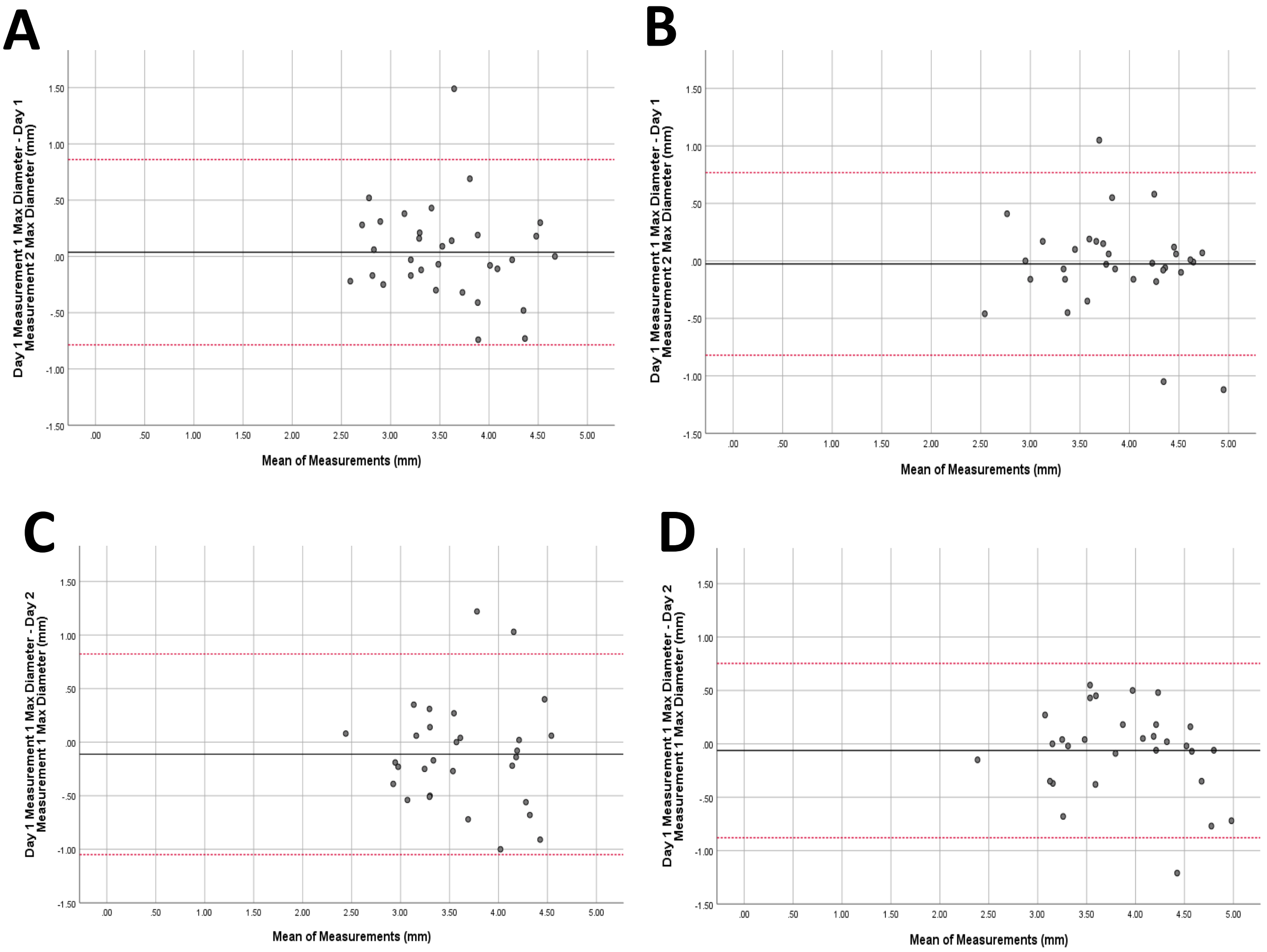

Bland-Altman plots of maximum diameters for manual and automated analysis. A: Automated results maximum diameters on the same day, B: Manual results maximum diameters on the same day, C: Automated results maximum diameters on separate days, D: Manual results maximum diameters on separate days, Broken red lines = 95% limits of agreement, % FMD = percentage flow mediated dilation.
